# Paradoxical Impact of Two Folate Receptors, FRα and RFC, in Ovarian Cancer: Effect on Cell Proliferation, Invasion and Clinical Outcome

**DOI:** 10.1371/journal.pone.0047201

**Published:** 2012-11-07

**Authors:** Michelle K. Y. Siu, Daniel S. H. Kong, Hoi Yan Chan, Esther S. Y. Wong, Philip P. C. Ip, LiLi Jiang, Hextan Y. S. Ngan, Xiao-Feng Le, Annie N. Y. Cheung

**Affiliations:** 1 Department of Pathology, University of Hong Kong, Hong Kong, Special Administrative Region of China; 2 Department of Obstetrics and Gynaecology, University of Hong Kong, Hong Kong, Special Administrative Region of China; 3 Department of Experimental Therapeutics, Division of Cancer Medicine, University of Texas M. D. Anderson Cancer Center, Houston, Texas, United States of America; Queensland University of Technology, Australia

## Abstract

Despite being an essential vitamin, folate has been implicated to enhance tumor growth, as evidenced by reports on overexpression of folate receptor alpha (FRα) in carcinomas. The role of another folate transporter, reduced folate carrier (RFC), is largely unknown. This study investigated the roles of folate, FRα and RFC in ovarian cancers. We demonstrated FRα mRNA and protein overexpression and reduced RFC expression in association with FRα gene amplification and RFC promoter hypermethylation, respectively. FRα overexpression was associated with tumor progression while RFC expression incurred a favorable clinical outcome. Such reciprocal expression pattern was also observed in ovarian cancer cell lines. Folate was shown to promote cancer cell proliferation, migration and invasion *in vitro*, and down-regulate E-cadherin expression. This effect was blocked after either stable knockdown of FRα or ectopic overexpression of RFC. This hitherto unreported phenomenon suggests that, RFC can serve as a balancing partner of FRα and confer a protective effect in patients with high FRα-expressing ovarian carcinomas, as evidenced by their prolonged overall and disease-free survivals. In conclusion, we report on the paradoxical impact of FRα (putative oncogenic) and RFC (putative tumor suppressive) in human malignancies. FRα and RFC may potentially be explored as therapeutic target or prognostic marker respectively. We recommend caution and additional research on folate supplements in cancer patients.

## Introduction

Ovarian carcinomas account for the highest mortality amongst all gynecological cancers in the world [1], [2]. While the incidence of ovarian carcinomas varies between different ethnic groups, its incidence in Asian countries is on a rising trend [3], [4]. The reasons for this remain largely unknown or controversial. Several lifestyle risk factors have been implicated. These include diet, obesity, fertility and parity statuses. On the other hand, it has became a general belief that high intake of micronutrients such as folate, vitamin C, vitamin E may protect against cancers [5]. As such, better understanding of the effects of nutritional elements on carcinogenesis is important to improve the strategies for cancer prevention and management.

Folate is a water-soluble B vitamin found in most vegetables. A high dietary folate intake has been reported to associate with a lower risk of developing ovarian cancers, in particular, those who consume alcohol [6], [7], [8], [9]. It is closely related to its function on DNA synthesis and its involvement in the related methionine metabolic pathway essential for DNA methylation. Folate deficiency would therefore leads to DNA hypomethylation, altered gene expression and the misincorporation of uracil into DNA, leading to chromosome damage, all of which, are key factors for carcinogenesis [10], [11]. It would appear that folate is an important vitamin essential in normal functioning of cells, and to prevent the initiation of cancer. However, there is increasing evidence to show that folate may in fact enhance cancer progression in established carcinomas of colon and rectum, breast and prostate [12], [13], [14].

Folate uptake involves several transporters, such as folate receptors, and reduced folate carrier (RFC) [15], [16]. Folate receptor alpha (FRα), a single chain glycosyl-phosphatidylinositol–anchored membrane protein, enhances folate uptake through endocytosis. Its overexpression has been reported in ovarian cancers, implying that it may promote tumor growth [17], [18], [19], [20]. RFC is an ubiquitously expressed transporter for natural folates and classical antifolates, and can control folate uptake in a bi-directional manner [16]. Loss of RFC with subsequent effects of folate deficiency was found to promote cancer progression in colorectal cancer [16], [21]. It would therefore be logical to assume that these two folate transporters, FRα and RFC, exert different effects in cancer progression.

Although overexpression of FRα in ovarian cancers has been established, the expression status and functional roles of RFC remain largely unknown. In this study, we investigated the expression, genetic and epigenetic profiles of FRα and RFC in normal ovarian epithelium and ovarian cancer, and correlated with clinicopathological parameters. Their functional roles and possible downstream targets on cell proliferation, migration and invasion in relation to folate in ovarian cancer were also assessed. We endeavoured to better understand the roles of folate and its transporters in ovarian carcinogenesis, and explore the possible effects of folate intake in cancer patients.

## Materials and Methods

### Clinical Samples and cell lines

One hundred and fifty three formalin-fixed paraffin embedded samples of ovarian tumors, including 11 inclusion cysts/benign cystadenomas (22–63 years; mean age, 50 years), 19 borderline tumors (20∼46 years; mean age, 30 years), 83 carcinomas (34 to 83 years; mean 51 years) of different histological subtypes and 44 corresponding metastatic foci (Table 1), were collected from the Department of Pathology, Queen Mary Hospital, the University of Hong Kong. All patients underwent surgery and 67 patients with ovarian cancers were also treated with chemotherapy including platinum/paclitaxel. The follow-up period ranged from five to 209 months (mean 63 months). Thirty three randomly selected clinical samples of ovarian tumors and their corresponding normal counterparts, including fallopian tubes and/or contralateral ovaries, with available frozen blocks were also retrieved. Informed consent was obtained by all patients and the use of these clinical samples was approved by Institutional Review Board of the University of Hong Kong/Hospital Authority Hong Kong West Cluster (HKU/HA HKW IRB)(Institutional Review Board number: UW10-129). Haematoxylin Eosin stained sections of the frozen blocks of each sample were reviewed by two of us (A.N.Y.C. and P.P.C.I.) to confirm the diagnosis and to ensure that more than 80% tumor cells were present in the tumor blocks.

**Table 1:** Correlation of FRα and RFC immunoreactivities with different diagnostic categories and clinicopathological parameters in ovarian cancer.

Characteristics	Case (n)	FRα		RFC	
		Mean ± SD	*P*-value	Mean ± SD	*P*-value
**Diagnostic categories**					
Cysts/benign	11	1.75±1.30		5.36±3.11	
Borderline	19	3.96±2.47		4.50±3.06	
Carcinomas	83	7.10±3.25	<0.001 *	2.94±2.33	<0.008 *
Carcinomas‡	21	8.14±3.06		2.45±2.40	
Metastatic foci‡	44	7.60±3.18	0.522†	3.11±1.80	0.128†
**Stage (FIGO)**					
I	28	5.94±2.83		2.45±1.90	
II	12	8.35±3.36		3.38±1.98	
III	25	7.72±3.22		3.60±2.52	
IV	14	7.14±3.56	0.092*	2.57±2.87	0.188*
I	28	5.94±2.83		2.45±1.90	
II–IV	51	7.71±3.31	0.022 †	3.27±2.50	0.113†
**Histological grade**					
1	18	5.68±1.94		3.67±2.46	
2	38	7.21±3.39		2.61±2.16	
3	25	8.18±3.47	0.042 *	2.80±2.30	0.262*
Low (1)	18	5.68±1.94		3.67±2.46	
High (2–3)	63	7.60±3.43	0.022 †	2.68±2.20	0.123†
**Histology**					
Serous	30	8.53±3.30		3.23±2.85	
Clear Cell	20	5.13±2.74		2.50±2.43	
Endometrioid	26	7.60±2.83		3.02±1.73	
Mucinous	7	4.71±1.90	0.001 *	2.64±1.55	0.780*
Non-mucinous	76	7.32±3.27		2.97±2.39	
Mucinous	7	4.71±1.90	0.027 †	2.64±1.55	0.947†
**Chemosensitivity** §					
Sensitive	56	7.38±3.29		2.99±2.30	
Resistant	15	6.34±3.03	0.259†	2.50±2.21	0.564†

Intensity values are expressed as “Histoscores” as specified in Methods.

* Kruskal–Wallis rank test;

† Mann-Whitney test;

‡ Randomly selected primary carcinomas with matched metastatic foci.

§ Chemosensitive-patients remained disease free more than 6 months after completion of first-line chemotherapy.

Those with significant *P*-values are underlined.

Two immortalized ovarian epithelial cell lines, HOSE 6-3 and HOSE 17-1, and nine ovarian cancer cell lines, SKOV-3, OVCAR-3, OVCA 420, OVCA433, OC316, Dov13, ES-2, TOV21G, SW626 (ATCC; Manassas, VA) were cultured as previously described [22], [23].

### Real-time PCR (qPCR)

Total RNA from frozen clinical samples and cancer cell lines was extracted using Trizol reagent (Invitrogen). Genomic DNA contamination was removed by treating with DNase I (Invitrogen) treatment. 2.5 µg total RNA was reverse transcribed by SuperScript Reverse Transcriptase (Invitrogen, San Diego, CA). Genomic DNA was extracted using phenol/chloroform (Invitrogen). qPCR was performed with ABI Prism 7700 sequence detection system (Applied Biosystems, Foster City, CA) as described [22], [23], [24]. Primer sequences for evaluating mRNA expression of FRα, RFC and GAPDH (as internal control) were as follows: FRα sense, 5′- AAGTGCGCAGTGGGAGCT -3′, and antisense, 5′- CATTGCACAGAACAGTGGGTG -3′; RFC sense, 5′- CGAAACCTCGGCTTCGGAGC -3′, and antisense, 5′- GCACGTAGTAGACCACCAGG -3′; GAPDH sense, 5′- TCCATGACAACTTTGGTATCGTG -3′, and antisense, 5′- ACAGTCTTCTGGGTGGCAGTG -3′. The PCR purity was confirmed by gel electrophoresis. Primer sequences (sense, 5′- GTATGCATGGCTTCCTGCAGG -3′, and antisense, 5′- ACTTGTTAAACCCTGTAGAGAGG -3′) for evaluating FRα gene copy number were designed based on the genomic sequence of intron 3 and intron 4 of FRα (Ensemble database). TRAT1 was used as the reference gene [25]. Ovarian cancer samples having ≥ two-fold increase from their corresponding normal counterparts were considered as positive for FRα gene amplification.

### Immunoblotting

Cells were harvested with lysis buffer (0.125 M Tris, pH 6.8 at 22°C containing 1% NP-40 (v/v), 2 mM EDTA, 2 mM N-ethylmaleimide, 2 mM PMSF, 1 mM sodium orthovanadate and 0.1 µM sodium okadate], and cleared by centrifugation at 4°C. Protein concentration was determined by DC (detergent compatible) protein assay (Bio-Rad Laboratories, Hercules, CA). 20 µg protein was resolved by SDS-PAGE, transferred to polyvinylidene difluoride membrane, and hybridized with antibodies specific to FRα (1∶1000; Alexis Biochemical, San Diego, CA; ALX-804-439), RFC (1∶1000; Affinity BioReagents; Golden, CO; PA1-9553), E-cadherin (1∶5000; BD Biosciences; Palo Alto, CA; 610182), and actin (1∶1000; Sigma, St. Louis, MO; A5060) and appropriate secondary antibodies (Santa Cruz Biotechnology, Santa Cruz, CA). The blots were developed by Enhanced Chemiluminescence (ECL) Plus detection system (Amersham, Arlington Heights, UK), and visualized with X-ray film (Galen Medical Group, Chattanooga, TN) [22], [23], [24].

### Immunohistochemistry

Immunohistochemical staining was performed as described in earlier reports [22], [23], [24]. For FRα immunohistochemistry, paraffin sections were treated with goat anti-folate receptor antibody conjugated with horse radish peroxidase (1∶200; Abcam; Cambridge, MA; ab20572). Since the anti-FRα antibody from Alexis Biochemical used for immunoblotting failed to have satisfactory result on immunostaining using paraffin sections, another antibody from Abcam was used. Although this antibody may recognize other isoforms of FR, the beta and gamme isoforms of FR were reported to be predominantly expressed in placenta and hematopoetic cells but not in other tissues [26], [27]. This antibody can therefore be used to detect FRα immunoreactivity in ovarian cancers. For RFC immunohistochemistry, chicken anti-RFC antibody (1∶200; Affinity BioReagents) was applied, followed by biotin-rabbit anti-chicken IgG (H+L). 3-diaminobenzidine-hydrogen peroxide was used as chromogen. Microwave antigen recovery using citrate buffer (pH 6.0) was performed. Omission or substitution of the primary antibody with preimmune IgG serum was used as a negative control. Intensity in stained epithelial cells was scored as 0 (negative), 1 (faint), 2 (moderate), and 3 (strong). The percentage of stained cells was rated as 0 (<5%), 1 (5%–25%), 2 (26%–50%), 3 (51%–75%) and 4 (>75%). Immunoreactivity was assessed by multiplying the staining intensity by the percentage of stained cells to give a composite a composite “Histoscore” [22], [23]. High and low levels of FRα and RFC were defined by “HistoScores” cut off at mean.

### Demethylation treatment

SKOV-3 and OVCA420 cells were treated with 0, 5 or 10 µM 5-Aza-2′-deoxycytidine (5-aza-dc, a DNA methylation inhibitor) for 72 hours [28]. Control cells were treated with equal volume of dimethyl sulfoxide (DMSO). Total RNA was extracted from cells. The transcription activity of RFC was determined by qPCR.

### DNA preparation, bisulfite treatment and methylation-specific PCR (MSP) analysis

Genomic DNA from frozen clinical samples was extracted using phenol/chloroform. Bisulfite treatment was performed as described [28]. Primers specific to the methylated (sense, 5′- TTCGTCGTAGTTTGCGAATG -3′, and antisense, 5′- CAACACGTACCTAAACGCGA -3′) and unmethylated (sense, 5′- TTTGTTGTAGTTTGTGAATGG -3′, and antisense, 5′- ACAACACATACCTAAACACAA -3′) RFC promoter A were reported previously [29]. The annealing temperature was 52°C and 56°C for methylated and unmethylated promoter A respectively. MSP products were detected by electrophoresis on 2% agarose gel with ethidium bromide staining. Normal lymphocyte DNA methylated with Sssl methyltransferase was used as positive control. Untreated genomic DNA and water blanks without DNA were used as negative controls.

### Stable knockdown of FRα, ectopic overexpression of RFC and folate treatment in SKOV-3

SKOV-3, an ovarian cancer cell line with relatively high FRα and low RFC expression, was used. To stable knockdown FRα, cells were transfected with a set of shRNA constructs against human FRα, pRS-sh FRα (Origene, Rockville, MD), selected with puromycin (1.5 µg/ml) [22], [23], [24]. The pRS vector was used as controls. To transient overexpress RFC, pcDNA3-RFC plasmid (kindly provided by Prof L Matherly, Michigan Cancer Foundation) and the empty pcDNA3 vector (control) was transfected into control SKOV-3 cells using Lipofectamine 2000 (Invitrogen) [22], [23], [24]. Cells were cultured in Medium 199 (Invitrogen)/MCDB 105 (Sigma) medium containing 22.7 nM folic acid and supplemented with 10% fetal bovine serum (FBS) (JRH Biosciences, Lenexa, KS) [22], [23]. shFRα cells and RFC overexpressing cells (2 days after transfection) were pretreated with folate-free RPMI 1640 medium (Invitrogen) supplemented with 10% dialyzed FBS containing 0.6 nM folic acid (Invitrogen) for 2 days, trysinized, counted, plated for functional assays and then treated with different doses of folic acid, a synthetic folate, including 0, 6, 12 and 60 nM. The folic acid concentrations used are based on the physiological range in plasma, which ranges from <7 nM in individuals with a negative folate balance to >50 nM in individuals with >400 µg/d of folate consumption [30], which is the estimated folate intake by supplement nonusers in North America [13]. The folic acid deficiency concentration selected (12 nM) was based on the observation that such concentration is the lowest requirement for cell growth [31]. Protein was extracted 2 days after treatment.

### MTT (3-[4,5-dimethylthiazol-2-yl]-2,5-diphenyl tetrazolium bromide) assay

Cell proliferation was determined by MTT assay (Sigma) as described [22], [24]. Cells were seeded in 96-well plates with 2000 cells/well. At specific time points, 10 µl MTT was added to each well. Plates were incubated at 37°C for 4 h, followed by addition of 100 µl DMSO to each well for dye extraction. Cell proliferation was determined by measuring the absorbance of samples at 570 nm with 630 nm as the reference wavelength.

### *In vitro* migration and invasion assays

*In vitro* migration and invasion assays were performed as described [22], [23], [24]. 1.25×10^5^ cells were plated on the upper side of a Transwell insert and allowed to migrate through an 8-µm pore size membrane (migration assays) or invade through a Matrigel–coated membrane (invasion assays). Cells at the upper side of the membrane were removed and the migrated or invaded cells were fixed with methanol, stained with 0.5% crystal violet, and counted under a light microscope in 5 random fields after 24 h or 48 h respectively.

### Statistical Analysis

Statistical analysis was performed using SPSS 15.0 for Windows (SPSS Inc., Chicago, IL). Mann-Whitney test was used for comparison between two groups whereas Kruskal–Wallis rank test was used for comparison among multiple groups. Survival analysis was performed by Kaplan–Meier analysis and log-rank test. Cox regression analysis was used for multivariate survival analysis. *P* values<0.05 were considered as statistically significant.

## Results

### Overexpression of FRα was associated with ovarian tumor progression

**Figure 1:**
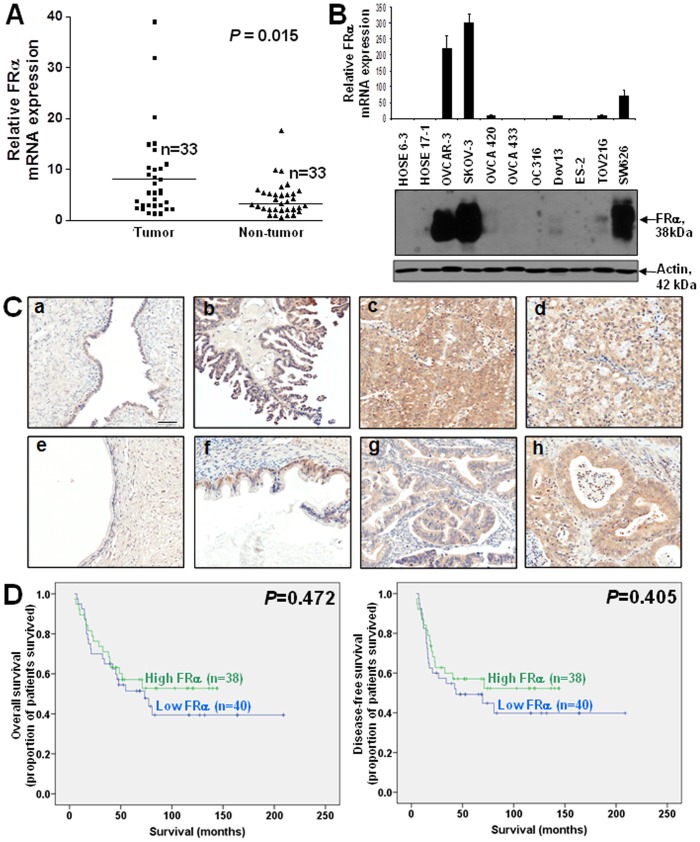
Overexpression of FRα in ovarian cancer. (A) qPCR analysis of FRα mRNA in ovarian tumors and the corresponding non-tumor counterparts. (B) mRNA (upper panel) and protein (lower panel) expression of FRα in two immortalized ovarian epithelial cell lines, HOSE 6-3 and HOSE-17-1, and nine ovarian cancer cell lines, OVCAR-3, SKOV-3, OVCA 420, OVCA433, OC316, Dov13, ES-2, TOV21G, SW626, as assessed by qPCR and immunoblotting respectively. (C) Immunoreactivity of FRα in serous (a) and mucinous (e) benign ovarian cystadenomas, serous (b) and mucinous (f) borderline ovarian tumors and serous (c), mucinous (g), clear cell (d) and endometrioid (h) ovarian carcinomas. Scale bar = 100 µm. (D) Kaplan-Meier overall (left panel) and disease-free (right panel) survival curves for ovarian cancer patients with high and low levels of FRα (cut off at mean).

By qPCR, significantly higher FRα mRNA was found in cancer samples when compared with the corresponding non-tumor counterparts after normalization with GAPDH (*P* = 0.015) (Figure 1A). By immunohistochemistry, strong FRα immunoreactivity was observed in ovarian cancers in contrast to moderate staining of FRα in borderline tumors and weak or absence of staining in benign cystadenomas/inclusion cysts (Figure 1C). Indeed, significantly higher FRα immunoreactivity was detected in ovarian cancers and borderline tumors than in benign cystadenomas/inclusion cysts (all *P*<0.05, Table 1). At cell lines level, six out of nine ovarian cancer cell lines also showed up-regulation of FRα mRNA and protein expression with SKOV-3, OVCAR-3 and SW626 showing strong expression while OVCA 420, Dov13 and TOV21G showed weak expression when compared with two normal ovarian epithelium cell lines in which no FRα mRNA and protein expression was detected (Figure 1B).

In clinical samples, high FRα mRNA expression and immunoreactivity were found to be significantly associated with advanced stages of disease and poor histological grade, factors associated with tumor aggressiveness (Tables 1 and 2). However, no significant difference of FRα immunoreactivity was found between the chemosensitive and chemoresistant cases (Table 1). Kaplan-Meier-survival analyses also did not reveal association between high FRα expression and overall or disease-free survival (Figure 1D).

**Table 2:** Correlation between clinicopathological parameters and mRNA expression of FRα and RFC in ovarian cancers.

	FRα			RFC		
Characteristics	mRNA expression			mRNA expression		
	Normal (no. of cases)	Increased (no. of cases)	*P*-value*	Normal (no. of cases)	Decreased (no. of cases)	*P*-value*
**Stage (FIGO)**						
I	11	5		4	12	
II	1	2		1	2	
III	2	9		2	9	
IV	2	1	0.062	1	2	0.920
Early (I)	11	5		4	12	
Late (II–IV)	5	12	0.024	4	13	1.000
**Histological grade**						
1	1	1		0	2	
2	11	5		5	11	
3	4	11	0.064	3	12	0.545
Low (1–2)	12	6		5	13	
High (3)	4	11	0.037	3	12	0.699
**Histology**						
Serous	3	9		2	10	
Endometrioid	5	6		4	7	
Clear Cell	4	2		1	5	
Mucinous	4	0	0.052	1	3	0.695
Non-mucinous	12	17		7	22	
Mucinous	4	0	0.044	1	3	1.000
Serous	3	9		2	10	
Non-serous	13	8	0.071	6	15	0.443

* Fisher's exact test. Those with significant *P*-values are underlined.

### RFC was down-regulated in ovarian cancers and correlated with good prognosis of patients

**Figure 2:**
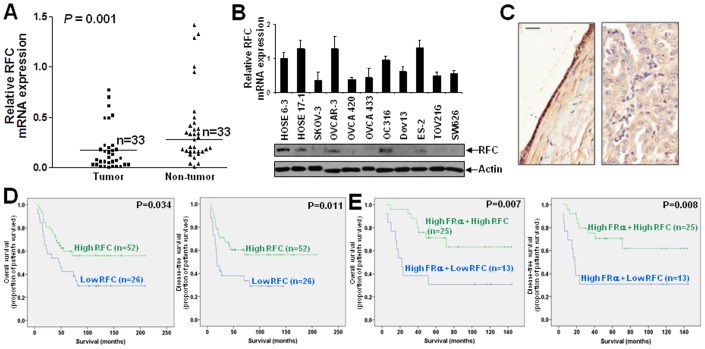
Down-regulation of RFC in ovarian cancers and correlation with prognosis of patients. (A) qPCR analysis of RFC mRNA in ovarian tumors and the corresponding non-tumor counterparts. (B) mRNA (upper panel) and protein (lower panel) expression of RFC in two immortalized ovarian epithelial cell lines, HOSE 6-3 and HOSE-17-1, and nine ovarian cancer cell lines, SKOV-3, OVCAR-3, OVCA 420, OVCA433, OC316, Dov13, ES-2, TOV21G, SW626, as assessed by qPCR and immunoblotting respectively. (C) Immunoreactivity of RFCin inclusion cyst (a) and serous ovarian carcinomas (b). Scale bar = 100 µm. (D) Kaplan-Meier overall (left panel) and disease-free (right panel) survival curves for ovarian cancer patients with high and low levels of RFC (cut off at mean). (E) Kaplan-Meier overall (left panel) and disease-free (right panel) survival curves for high FRα expressed ovarian cancer patients with high and low levels of RFC (cut off at mean).

In contrast to FRα, ovarian cancer samples displayed significantly lower RFC mRNA when compared with the corresponding non-tumor counterparts as assessed by qPCR (*P* = 0.001) (Figure 2A). Immunohistochemical analysis also revealed strong RFC immunoreactivity in inclusion cysts/benign cystadenomas and weak expression in ovarian cancers (Figure 2C). Six out of nine ovarian cancer cell lines also displayed down-regulation of RFC mRNA and protein expression when compared with two normal ovarian epithelium cell lines (Figure 2B).

RFC mRNA and immunoreactivity did not correlate with stages of disease, histological grade and histological subtypes (Tables 1 and 2). Interestingly, there was a significant association between low expression of RFC, and shorter overall (*P* = 0.034) and disease-free (*P* = 0.011) survival (Figure 2D). Moreover, among ovarian cancers with high FRα expression, the overall (*P* = 0.007) and disease-free (*P* = 0.008) survival was significantly longer in those with high RFC expression (Figure 2E).

### FRα gene amplification and RFC promoter methylation contributed to dysregulated gene expression in ovarian cancers

By qPCR, 11 out of 33 (33.3%) cancer samples displayed FRα gene (FOLR1, chromosome 11q13.3) amplification when compared with the corresponding non-tumor counterparts. All amplified cases showed elevated mRNA expression. FRα amplification was correlated with its mRNA expression (*P*<0.001, Fisher's exact test) (Table 3).

**Table 3:** Correlation of FRα amplification and RFC promoter methylation with their mRNA expression in ovarian cancers.

FRα		Gene amplification		*P*-value (Fisher's exact test)
		Non-amplified (no. of cases)	Amplified (no. of cases)	
**mRNA expression**	**Normal (no. of cases)**	16	0	<0.001
	**Increased (no. of cases)**	6	11	

RFC		Promoter DNA methylation		*P*-value (Fisher's exact test)
		Unmethylated (no. of cases)	Methylated (no. of cases)	
**mRNA expression**	**Normal (no. of cases)**	8	0	0.005
	**Decreased (no. of cases)**	11	14	

**Figure 3:**
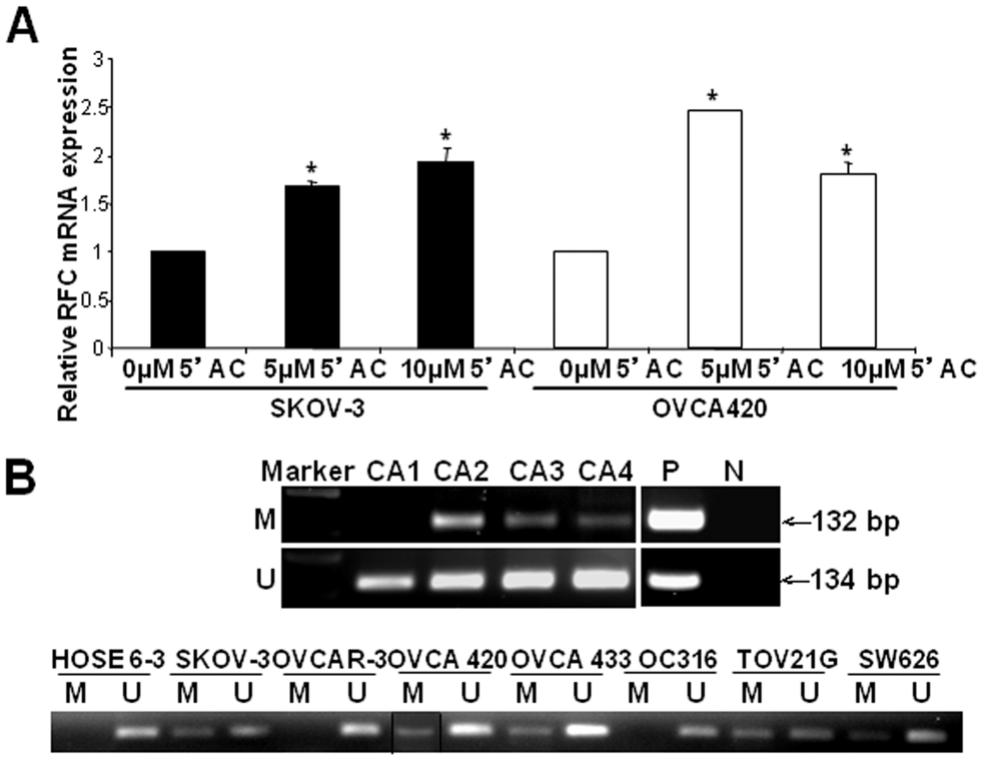
Promoter hypermethylation of RFC gene in ovarian cancers. (A) The relative mRNA expression of RFC in SKOV-3 and OVCA 420 after 5-aza-dc treatment with indicated concentrations for 72 hours. Each experiment was performed in triplicate. Bars, means of fold change ±SD. *, *P*<0.05. (B) Representative ovarian cancers (CA) (upper panel) and ovarian cell lines (lower panel) of MSP on RFC methylation status. M, DNA marker; P, positive control; N, negative control; M, methylated alleles; U, unmethylated alleles.

On the other hand, reduced expression of RFC in ovarian cancers was related to hypermethylation. After treatment of SKOV-3 and OVCA420 ovarian cancer cells by 5-aza-dc, a DNA methylation inhibitor, two-fold and 2.5-fold increase of RFC gene expression was detected respectively (Figure 3A). Furthermore, promoter hypermethylation of RFC gene (chromosome 21q22.2) was found in 14 out of 33 (42.4%) ovarian cancer samples by MSP. Representative examples of MSP were shown in Figure 3B. In contrast, only 3 out of 33 (9%) of non-tumor samples showed hypermethylation. Unmethylated alleles were detected in all tumor and non-tumor samples. Promoter hypermethylation of RFC significantly inversely correlated with its mRNA expression (*P* = 0.005, Fisher's exact test) (Table 3). By MSP, we also detected RFC promoter hypermethylation in five ovarian cancer cell lines SKOV-3, OVCA 420, OVCA433, TOV21G and SW626 (Figure 3B), all of them showed down-regulated RFC mRNA and protein expression (Figure 2B). In contrast, no methylated alleles were detected (Figure 3B) in the normal ovarian epithelium cell line HOSE 6-3 and two cancer cell lines OVCAR-3 and OC316, which displayed RFC mRNA and protein expression (Figure 2B),

### Knockdown of FRα altered folate-mediated cell proliferation in SKOV-3 cells

**Figure 4:**
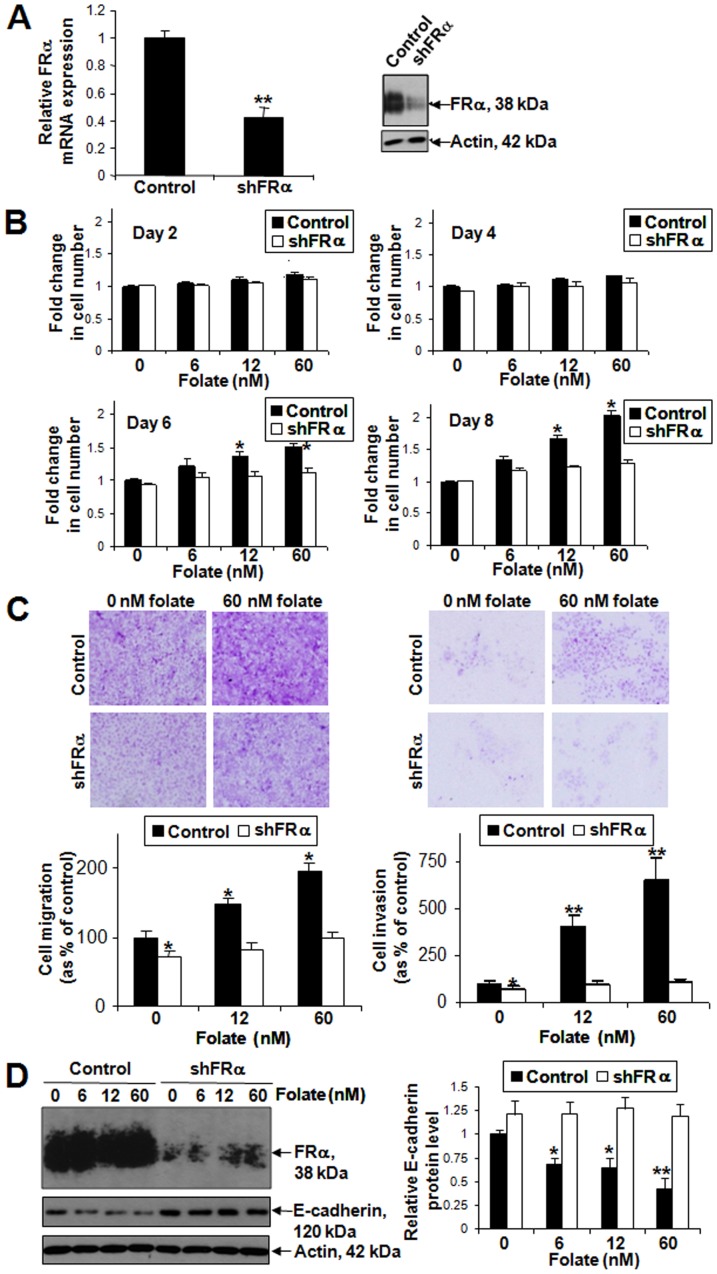
Folate induced SKOV-3 cell proliferation, migration and invasion and down-regulated E-cadherin through FRα. (A) Stable knockdown of FRα mRNA and protein in SKOV-3 as detected by qPCR (left panel) and immunoblotting (right panel) respectively. **, *P*<0.005. (B) Cell proliferation rate of control and shFRα SKOV-3 cells treated with 6, 12 and 60 nM folate at 2, 4, 6 and 8 days displayed as fold change relative to control without folate treatment (0 nM). n = 3; *, *P*<0.05. (C) *In vitro* migration (left panel) and invasion assays (right panel) in control and shFRα SKOV-3 cells treated with 0, 12 and 60 nM folate using Transwell membrane without or with Matrigel coating respectively. Upper panels: representative images of migrating or invading SKOV-3 cells. Lower panels: Cell migration or invasion from SKOV-3 presented as percentage of control treated with 0 nM folate; n = 3; *, *P*<0.05; **, *P*<0.005. (D) Immunoblotting on FRα and E-cadherin using protein lysates prepared from control and shFRα SKOV-3 (left panel). Relative E-cadherin protein level as analyzed by ImageJ software (US National Institutes of Health); n = 3; *, *P*<0.05; **, *P*<0.005 (right panel).

After confirming the specific knockdown of FRα mRNA and protein expression in SKOV-3 cells (Figure 4A), we first determined the effects of FRα on folate-mediated cell proliferation by MTT assay. On days 2 and 4, no significant change of cell proliferation was found in control and shFRα SKOV-3 cells after folate treatment. By day 6, control cells showed proliferation in 12 and 60 nM folate treatment. On Day 8, 6, 12 and 60 nM folate-treated control cells showed does-dependent proliferation. In contrast, knockdown of FRα blocked folate-mediated cell proliferation (Figure 4B).

### Folate through FRα induced SKOV-3 cell migration and invasion and down-regulated E-cadherin

Next, we tested the effect of folate and FRα on SKOV-3 cell migration and invasion. Based on the effects of folate on cell proliferation, 12 and 60 nM doses were chosen for treating control and shFRα SKOV-3 cells. Transwell migration and invasion assays showed that 12 and 60 nM folate significantly induced cell migration and invasion in control cells whereas knockdown of FRα blocked folate-mediated cell migration and invasion (Figure 4C). We then determined the possible downstream target for folate mediated effect on cell migration and invasion. The expression of E-cadherin, an important cell–cell adhesion molecule essential for regulating cell motility, was found to be reduced does-dependently after folate treatment (Figure 4D). Such down-regulation of E-cadherin after folate treatment was also abrogated after knockdown of FRα.

### Ectopic overexpression of RFC in high FRα-expressing SKOV-3 counteracted folate-mediated cell proliferation, migration and invasion and restored E-cadherin expression

**Figure 5:**
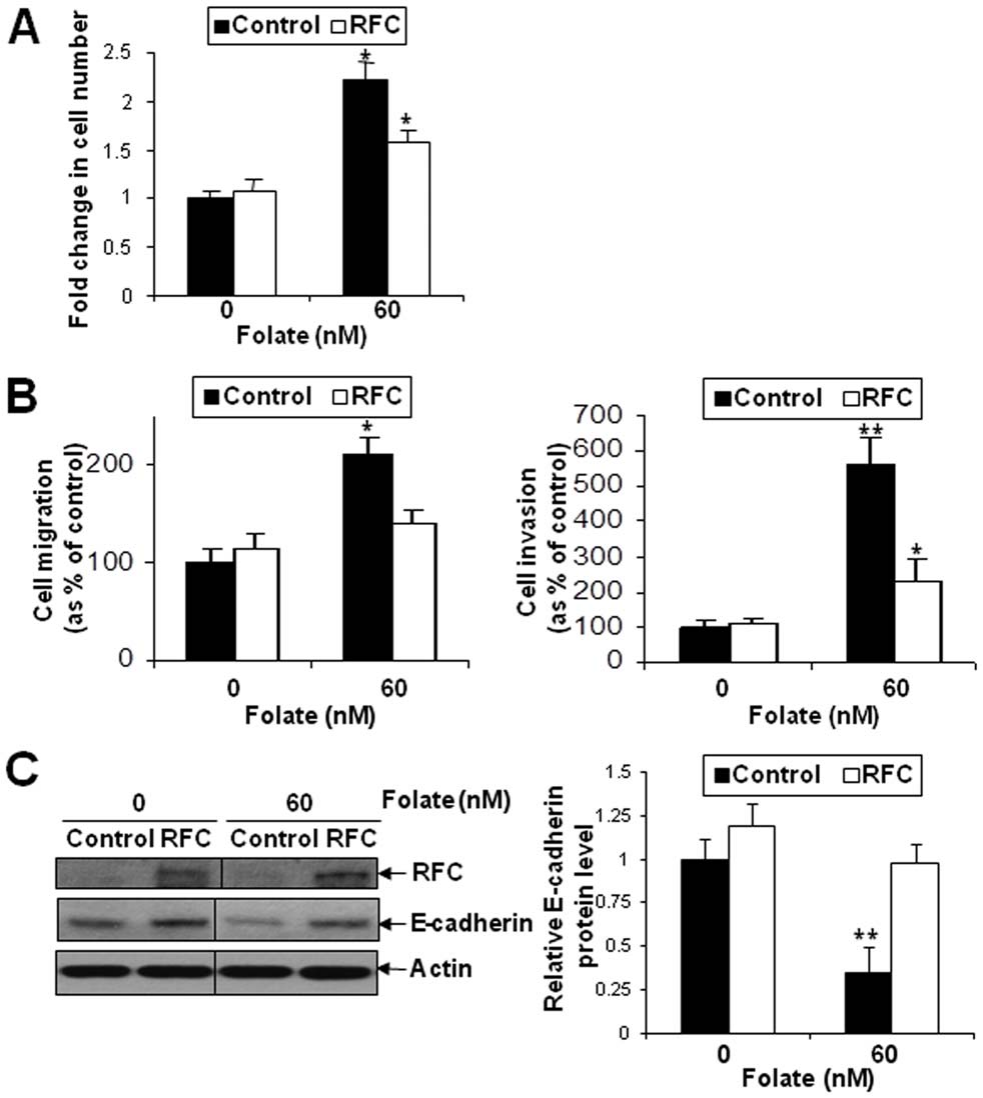
Ectopic overexpression of RFC in FRα-positive SKOV-3 counteracted folate-mediated cell proliferation, migration and invasion, and down-regulation of E-cadherin. (A) Cell proliferation rate of SKOV-3 cells with ectopically expressed RFC or control vector (control) treated with 60 nM folate after 5 days displayed as fold change compared to control without folate treatment (0 nM); n = 3; *, *P*<0.05. (B) *In vitro* migration (left panel) and invasion assays (right panel) in SKOV-3 cells with ectopically expressed RFC or control vector treated with 0 and 60 nM folate displayed as percentage of control treated with 0 nM folate; n = 3; *, *P*<0.05; **, *P*<0.005. (C) Immunoblotting of RFC and E-cadherin using protein lysates prepared from SKOV-3 cells with ectopically expressed RFC or control vector (left panel). Relative E-cadherin protein level as analyzed by ImageJ software (US National Institutes of Health); n = 3; **, *P*<0.005 (right panel).

We have demonstrated overexpression of FRα and reduced expression of RFC in ovarian cancers, suggesting that they may exert opposite roles in the progression of ovarian cancer. More importantly, in patients with high FRα, the overall and disease-free survival was significantly longer in those with high RFC expression, implicating the protective role of RFC in high FRα cancers. To elucidate such protective role, *in vitro* functional studies were performed on FRα-positive SKOV-3 cells with ectopically expressed RFC after folate treatment. RFC was found to counteract folate-mediated cell proliferation (Figure 5A), migration and invasion (Figure 5B). Moreover, down-regulation of E-cadherin in cells after folate treatment was also abrogated after overexpressing RFC (Figure 5C).

## Discussion

In this study, we demonstrated the progressive increase in FRα mRNA and protein expression from non-tumor tissues, benign and borderline tumors to carcinomas. In addition, FRα gene amplification as a possible mechanism of its overexpression was also demonstrated for the first time in ovarian cancers. Overexpression of FRα in ovarian cancers [19], [20] as well as in cancers of kidney, lung and breast have been previously reported [32]. Our results also indicate that a high FRα expression correlates with poor histological grade and advanced stages of disease, suggesting its roles in ovarian tumor progression.

In contrast to FRα, a lower RFC mRNA and protein expression in ovarian cancers was found when comparing with normal tissues or benign tumors. RFC is ubiquitously expressed in normal tissue and is the major folate transport system for transporting natural folates, such as 5-methyl or 5-formyl tetrahydrofolate (THF), and antifolates such as methotrexate (MTX) and pemetrexed [16]. 5-methyl THF is a cofactor essential for DNA methylation which normally leads to the suppression of oncogenes [33]. Thus, loss of RFC has been described to contribute to colonic carcinogenesis [21]. In this study, we found that reduced expression of RFC was significantly associated with shorter overall and disease-free survivals, suggesting that RFC may be considered as a marker for good prognosis in ovarian cancer patients. Moreover, among patients with high FRα expressing ovarian cancers, the overall and disease-free survival was significantly better in those with high RFC expression than those without, implicating the protective role of RFC for patients with these tumors.

We also demonstrated that FRα amplification and RFC promoter methylation correlated with mRNA expression in ovarian cancers. In earlier reports, RFC promoter methylation has been found in breast cancer cells [34] and primary lymphomas [35]. Our findings suggested that up-regulation of FRα (a putative oncogenic folate transporter) and down-regulation of RFC (a putative tumor suppressor type folate transporter) were controlled genetically and epigenetically respectively during ovarian cancer development.

As noted in the introduction above, folate is essential for DNA synthesis [12], [13], [14], thereby indirectly exerts its effect on cell proliferation. Overexpression of the FRα in NIH/3T3 cells has been reported to induce increased cell growth in vitro and in vivo [36]. Using folate at various dosages ranging from 12 nM (considered as dietary deficient in North America) to 60 nM (considered normal for supplement non-users), we were able to demonstrate cell proliferation in FRα-positive SKOV-3 cells [13]. Conversely, when the FRα was knockdown by shRNA approach, this folate-mediated cell proliferation in SKOV-3 cells was lost, confirming the fact that folate indeed transports through FRα during the process of ovarian cancer cell proliferation. Similarly, intracellular expression of anti-FR antibodies in ovarian cancer cells has been reported to exert growth inhibitory effects as shown by reduced colony formation in soft agar [37].

Besides its effects on cell proliferation, we also demonstrated for the first time that folate increased SKOV-3 cell migration and invasion, possibly through the downregulation of cell-cell adhesion molecule E-cadherin. This effect was abrogated after the knockdown of FRα. In ovarian cancer, reduced E-cadherin expression has been described in the metastases but not in the corresponding primary ovarian tumors [38]. Patients with such loss of E-cadherin expression were found to have significantly shorter survival [39]. Moreover, simultaneous expression of caveolin-1 and E-cadherin in ovarian cancer cells stabilized adherens junctions through inhibition of src-related kinases [40] whereas loss of E-cadherin enhanced ovarian cancer metastasis through up-regulation of α5-integrin [41]. In our study, we were able to show E-cadherin down-regulation in folated-treated SKOV-3 cells, suggesting that the folate-mediated enhancing effect on ovarian cancer cell migration and invasion probably acts through FRα via down-regulation of E-cadherin expression.

Interestingly, our study also demonstrates folate-mediated cell proliferation, migration, invasion and E-cadherin reduction in FRα-positive SKOV-3. Such effect is abrogated with ectopically expressed RFC, supporting its tumour suppressive effect in FRα-expressed cells. This in vitro finding further explains the in vivo finding that in patients with high FRα expressing ovarian cancers, the overall and disease-free survival was significantly longer in those with concomitant high RFC expression.

It has been suggested that vitamin supplements, including folate, is beneficial to health. Folate is usually taken as folic acid, its synthetic form, which is fortified in many food products. Supplementation is often believed to be of value to those who suffer from long-term illnesses. Nevertheless, the beneficial effect of folate supplement among cancer patients is controversial [12], [13], [14]. Earlier studies have shown that folate plays a dual role in colorectal, breast and prostate cancers [12], [13], [14]. Our results also indicated that folate may potentially enhance the progression and growth of ovarian cancer cells, in particular, those with high FRα and low RFC expressions. Although folate may prevent cancer initiation, once the neoplasia is established, it appears to enhance cancer progression. Should folate supplements, generally considered by the public as a healthy option, therefore be taken more cautiously? Additional studies to further explore the benefits or harmful effects of folate supplement in cancer patients are necessary.

In conclusion, we demonstrated that folate and FRα contribute to the progression and growth of ovarian cancer cells through the regulation of cell proliferation, migration and invasion. In contrast, RFC can serve as a balancing partner of FRα and seems to exert a protective role in ovarian cancer patients, conferring longer survival among patients with cancers that showed a high FRα expression status. We also demonstrated a mechanistic link between folate, FRα, RFC and E-cadherin. The potential of FRα and RFC as alternative molecular therapeutic target or prognostic marker in ovarian cancers should be further explored, respectively.
